# Natural history of changes in knee skin temperature following total knee arthroplasty: a systematic review and meta-analysis

**DOI:** 10.1038/s41598-023-33556-7

**Published:** 2023-04-26

**Authors:** Lilach Gavish, Leonid Kandel, Gurion Rivkin, S. David Gertz, Oshrit Hoffer

**Affiliations:** 1 Institute for Research in Military Medicine (IRMM) and Institute for Medical Research (IMRIC), Faculty of Medicine, The Hebrew University of Jerusalem and the Israel Defense Forces Medical Corps, POB 12272, 9112001 Jerusalem, Israel; 2 The Saul and Joyce Brandman Hub for Cardiovascular Research and the Department of Medical Neurobiology, Institute for Medical Research (IMRIC), Faculty of Medicine, The Hebrew University of Jerusalem, Jerusalem, Israel; 3 Department of Orthopedics, Hadassah Medical Center and Faculty of Medicine, Hebrew University of Jerusalem, Jerusalem, Israel; 4 Department of Electrical Engineering, Afeka Tel-Aviv Academic College of Engineering, Tel-Aviv, Israel

**Keywords:** Medical research, Musculoskeletal system

## Abstract

Patients undergoing total-knee arthroplasty (TKA) have transient increases in anterior knee skin temperature (ST) that subside as recovery progresses–except in cases of systemic or local prosthetic joint infections (PJI). This meta-analysis was designed to quantify the changes in knee ST following TKA in patients with uncomplicated recovery as a prerequisite for assessing the usefulness of thermal imaging for diagnosis of PJI. This meta-analysis (PROSPERO-CRD42021269864) was performed according to PRISMA guidelines. PUBMED and EMBASE were searched for studies reporting knee ST of patients that underwent unilateral TKA with uncomplicated recovery. The primary outcome was the weighted means of the differences in ST between the operated and the non-operated knees (ΔST) for each time point (before TKA, and 1 day; 1,2, and 6 weeks; and 3,6, and 12-months post-TKA). For this analysis, 318 patients were included from 10 studies. The elevation in ST was greatest during the first 2-weeks (ΔST = 2.8 °C) and remained higher than pre-surgery levels at 4–6 weeks. At 3-months, ΔST was 1.4 °C. It decreased to 0.9 °C and 0.6 °C at 6 and 12-months respectively. Establishing the baseline profile of knee ST following TKA provides the necessary first step for evaluating the usefulness of thermography for the diagnosis of post-procedural PJI.

## Introduction

The number of Total-knee arthroplasty (TKA) procedures in the US is projected to reach approximately 3.5 million by 2030^1^. Prosthetic joint infection (PJI) occurs in up to 2% of primary arthroplasties leading to significant morbidity often requiring complex, multi-disciplinary treatments^2^. Nonetheless, uniform criteria have not been established for the diagnosis of TKA-associated PJI. The various guidelines emphasize that the diagnosis should be multi-disciplinary and include not only clinical examination and serum analysis, but also microbial culture, intraoperative findings, biochemical studies, and appropriate histopathological and immune-histochemical analyses^3,4^. While there is strong evidence for the usefulness of blood tests, including white cell counts and erythrocyte sedimentation rate, as well as C-reactive protein, and interleukin-6, imaging modalities localized to the knee region, such as PET-CT and nuclear scan, have been considered to provide only limited supporting evidence for the presence of PJI^3^.

**Figure 1:**
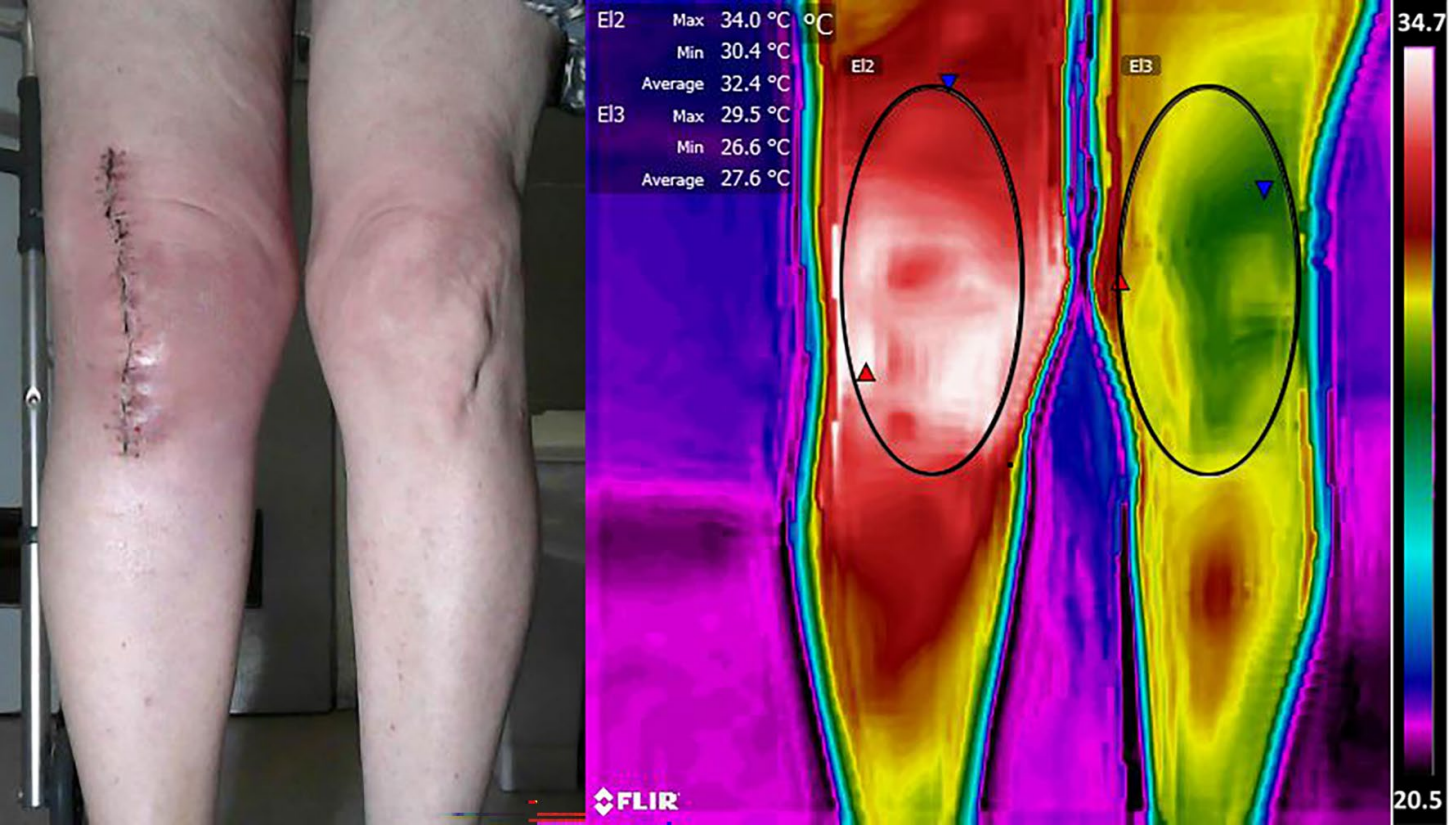
Thermal Imaging Following Total Knee Arthroplasty (TKA). **Left**: operated right knee 2wks post TKA; **Right**: thermal image showing elliptical area of measurement around the patella of the operated (R) and unoperated (L) knees. See temperature scale in the margin. Note increased skin temperature (red and white) of the operated knee. These images were from a clinical study (#0251-20-HMO) the PI of which was LK. Informed consent was obtained from the patients as required.

Infrared thermography is a noninvasive method that has been used in a variety of clinical entities to measure skin temperature distribution^5^. The latter is subtended by microcirculatory blood flow^6^ which is altered in inflammatory processes such as osteoarthritis^7,8^, infections^9^, or as a normal physiological response following surgical procedures. Incisions of the skin, muscle, and bone have been shown to be associated with an increase in C-reactive protein (CRP) and pro-inflammatory cytokines as well as local edema–all of which can contribute to increases in skin temperature (ST)^10,11^. Studies of patients undergoing TKA have shown a transient increase in knee ST that subsides as the recovery progresses (Fig. 1)^10,12–20^. However, this is not the case when there are systemic or prosthetic joint infections^9,21,22^.

The present study was designed, using a systematic-review and meta-analysis approach in order to establish the baseline, quantitative, temporal characteristics of knee skin-temperature, before and after TKA, in patients with uncomplicated recovery. This is a necessary prerequisite for assessing the usefulness of thermal imaging as an objective, non-invasive method for diagnosis of prosthetic joint infections.

## Results

**Figure 2:**
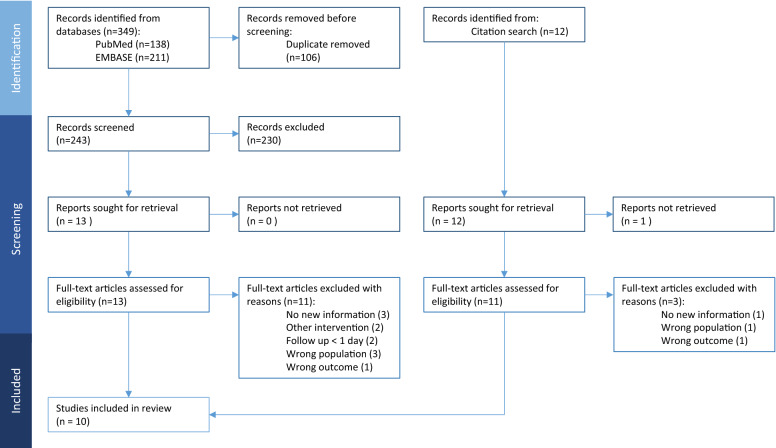
PRISMA Flow Chart. Preferred Reporting Items for Systematic Review and Meta-Analysis (PRISMA) flow diagram showing the identification, screening, eligibility and inclusion process.

The results of the systematic literature search are presented in the PRISMA flow diagram (Fig. 2). We identified 349 potentially relevant articles by systematically searching EMBASE and PubMed, and an additional 12 were obtained from manual citation search. After removal of duplicates, 243 records were screened of which 230 were excluded according to the eligibility criteria. Full texts of the remaining articles, together with those identified by citation research (n = 25), were sought for more detailed evaluation of which 10 were included in the meta-analysis (Table 1). In the studies with non-standard treatment (peripheral nerve block^13^, titanium nitride prosthesis coating^17^, and celecoxib [NSAID]^19^), only the control data were included. The studies were performed in Europe (UK, France, Italy, the Netherlands) and Asia (China and Thailand) and included patients with uncomplicated recovery the majority of which were women. The age of the patients ranged from 58 to 72 years. Of the 318 patients included in the systematic review (from 10 studies), the majority had a background of primary knee osteoarthritis (KOA) with the minority (20 patients) having other conditions including post-traumatic KOA, rheumatoid arthritis, osteonecrosis, and osteochondritis dissecans. TKA was performed by the standard medial approach in all studies. Not all studies reported the details of the TKA procedure. Of those that reported, 5 used a tourniquet, and one did not; 4 used the patellar component in the prosthesis, while 2 did not. The skin temperature of the anterior knee region was measured before TKA and from the first day to 5 years after the procedure. Skin temperature was measured using a surface infrared thermometer or a thermal imaging system (Table 1). Average skin temperature over the knee region was reported as absolute temperature (°C) of operated and non-operated knees or as the difference between the knees. The position of the patients at the time of temperature measurement varied between studies. In some, the knee temperature was measured when the patients were sitting^12,18^, some supine^13,15,17^, and, in one study, while standing ^16^. The acclimatization time varied between 3 and 20 min with some studies not reporting this important feature^10,13,14,19,23^. Regarding the measurement area, two groups that used thermal imaging measured the average temperature of the knee area surrounding the surgical scar including the patella^15,16^; while groups that used a thermometer mostly did not include the patella as part of the measurement^12^. By overall appraisal, three studies were graded as having good thermal methodology^12,16,18^, while all others were graded as ‘Fair’ (Table 2).

**Table 1:** Characteristics of Studies Included in the Meta-Analysis.

Author (Year)	Country (study period)	Sample size	M:F	Age (range)	Underlying condition	Tourniquet	Patellar component	Follow-Ups
Mehra^10^	UK (NR)	19	M:F 7:13	Age 72 (44–85)	18 KOA, 1 RA	Yes	Yes	1w, 6w, 3m
Haidar^12^	UK (2001–2004)	32	M:F 10:22	Age 70 (59–85)	KOA	Yes	Yes	1w, 6w, 3m, 6 m, 1y, 2y
Martinez^23^	France (NR)	20	M:F 1:19	Age 69	KOA	NR	NR	1d, 4d, 1m, 4m
Martin^13^	France (NR)	18*	M:F 4:14	Age 70	KOA	NR	NR	1d, 4d, 1w, 1m, 3 m
Honsawek^14^	Thailand (2007–2008)	49	M:F 9:40	Age 68 (50–78)	KOA	Yes	Yes	2w, 6w, 3m, 6 m
Romano^15^	Italy (2008)	40	M:F 28:12	Age 64 (53–78)	32 Primary KOA; 4 traumatic KOA, 4 osteonecrosis	NR	Yes	1d, 3d, 1w, 1m, 6w, 3m, 6 m, 1y
Mumingjiang^16^	China (2012–2013)	21	M:F 5:16	Age 58 (25–72)	15 Primary KOA; 3 post-traumatic KOA, 2 RA, 1 osteochondritis dissecans	Yes	NR	1d, 7d, 1m, 3m, 6m
van Hove^17^	Netherlands (2006–2007)	50*	M:F 13:37	Age 68	44 Primary KOA; 2 RA; 4 KOA + other	No	No	6w, 6m, 1y, 5y
Zeng^18^	China (2012–2014)	39	M:F 4:35	Age 67	KOA	NR	NR	1d, 3d, 5d, 1w, 2w, 1m, 3m, 6 m, 1y
Xu^19^	China (2016–2017)	30*	M:F NR	Age 64	KOA	Yes	No	1d, 3d, 1w, 2w, 6w

*Data from RCT control group; M:F, Male:Female counts; Age, mean [year old]; NR, not reported; d, day; w, week; m, month; y, year.

**Table 2:** Appraisal of Thermal Methodology.

Author (year)	Thermometry method	Thermal resolution	Patient position	Skin exposure time	Region of interest (ROI)	Appraisal	Comments
Mehra^10^	Thermometer	0.1 °C	No data	No data	Upper border of the patella—one inch on either side of the surgical wound. Average of the 2 readings	Fair	Description of thermal resolution was adequate, and ROI detailed; however, there was no description of patient position or exposure time
Haidar^12^	Thermometer	0.2 °C	Sitting (according to Fig. 2)	≥ 15 min	Anterior knee divided into 9 regions, with the patella in the center. The supero-medial region was used for measurements	Good	
Martinez^23^	Thermometer	No data	No data	No data*	Middle of patella	Fair	Device resolution could not be discerned. There was no description of patient position. *Other measurements required long exposure times, and it can be assumed that it was greater than 5 min
Martin^13^	Thermometer	No data	Supine	No data	Middle of patella. Average of 2 readings	Fair	Device resolution could not be discerned. Skin exposure time was not available
Honsawek^14^	Thermometer	No data	No data	No data	Anterior knee divided into 4 regions that diagonally border with the patella (supero-lateral, supero-medial, infero-lateral, and infero-medial). Average of 2 readings	Fair	There was no description of the device, patient position, or exposure time. The authors noted that measurements were taken at the same time of day, and each patient was measured with the same thermometer throughout the study
Romano^15^	Thermal imaging	0.1 °C	Supine	3 min	Anterior aspect. Elliptical area: major axis = 10 cm proximal and distal to the surgical wound; minor axis = approximately 12 cm in length crossing the midline of the major axis	Fair	At least 5 min were required to allow sufficient skin acclimatization
Mumingjiang^16^	Thermal imaging	0.5 °C	Standing, legs slightly apart	≥ 10 min	Anterior aspect. Rectangular area (15 cm × 10 cm) based on the surgical site	Good	
van Hove^17^	Thermometer	1 °C	Supine	4–5 min	Anterior aspect	Fair	The device resolution was low, and acclimatization time was borderline
Zeng^18^	Thermometer:	0.2 °C	Seated	15 to 20 min	Anterior aspect divided into patella and 4 regions (i.e. supero-lateral, supero-medial, infero-lateral and infero-rmedial border of the patella). Average of all regions	Good	
Xu^19^	Thermometer	0.2 °C	No data	No data	See Zeng	Fair	There was sufficiently detailed measurement methodology, but the position and exposure time were not reported

**Figure 3:**
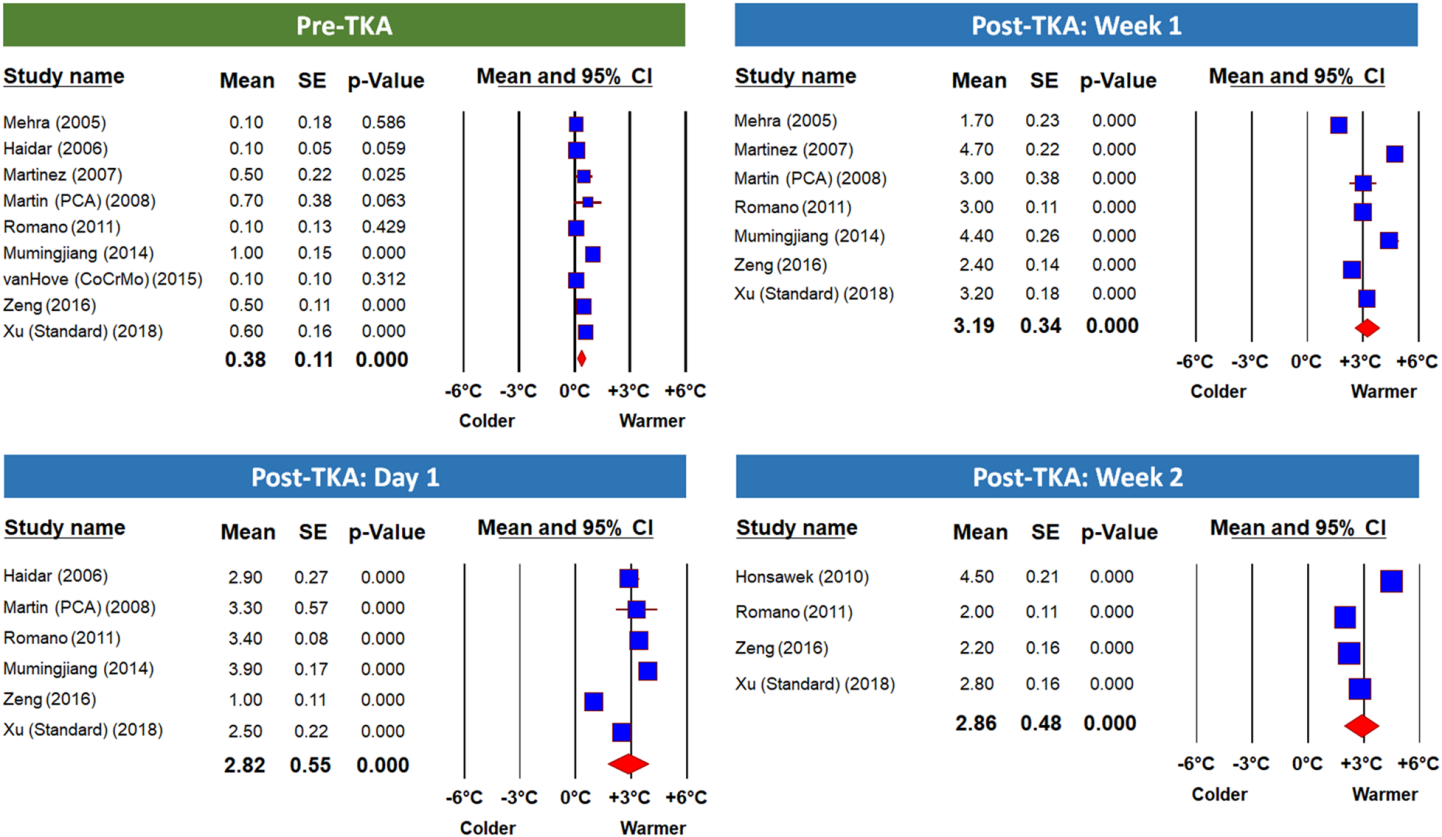
Meta-Analysis Forest Plots of ΔST Before TKA and at Early Follow Ups. This panel presents Forest plots of the mean temperature differences between the operated and non-operated knees (ΔST) before and up to 2 weeks post TKA using the random-effect model. Articles in each Forest plot are listed by year of publication. The mean, standard error and *p*-value of each study, and the pooled mean difference, are presented. Note that the difference in temperature between knees is significantly greater at all time points—even before the surgery.

**Figure 4:**
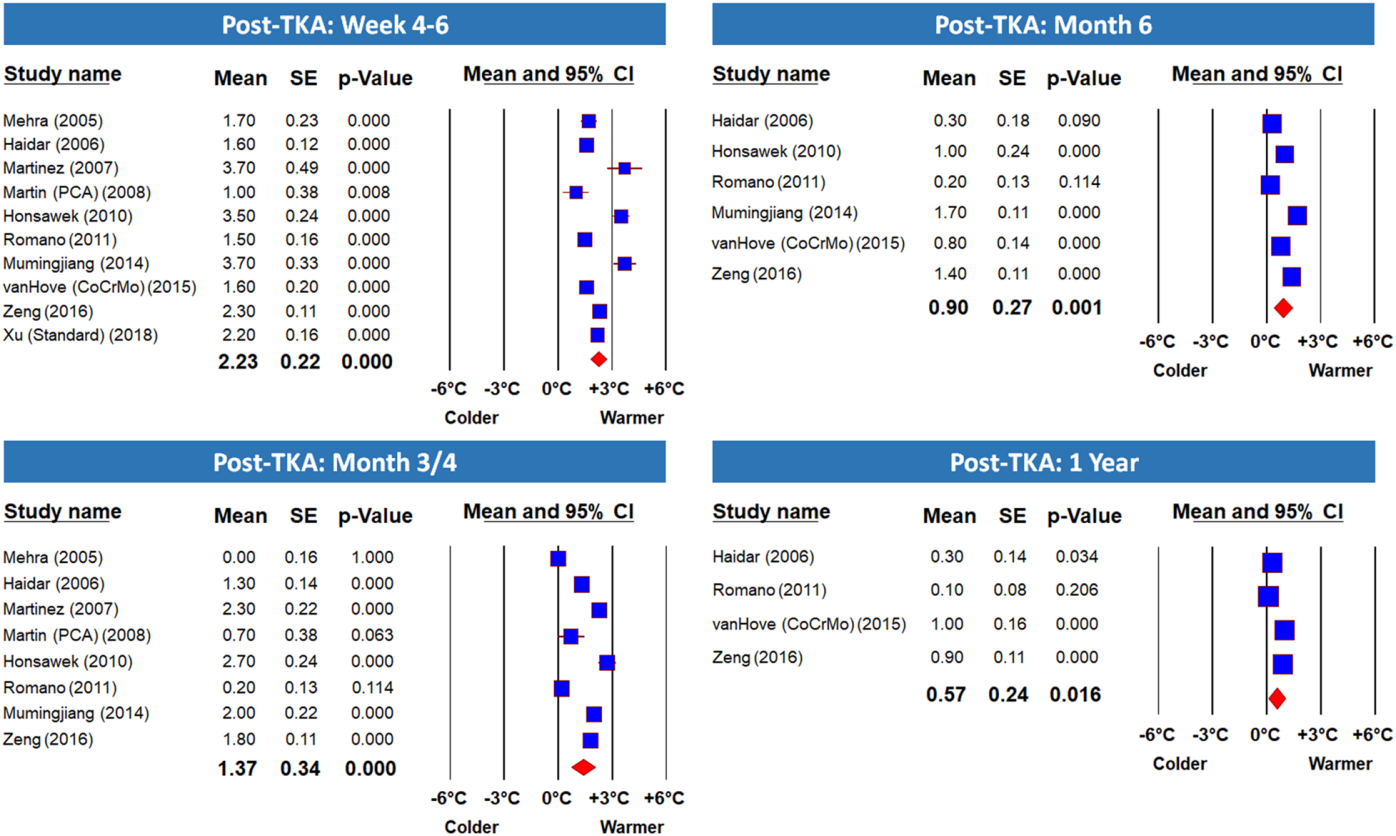
Meta-Analysis Forest Plots of ΔST at Medium and Long-Term Follow Ups. This panel presents Forest plots of ΔST from 4 to 6 weeks to 1 year post TKA. See details in the previous figure. Here too note that the operated knee was significantly warmer than the non-operated knee at all time points—even 1 year post-TKA.

Figures 3 and 4 present the results of the meta-analysis as 8 separate Forest Plots-one for each time point. These results are also summarized in Table 3 together with the statistics for heterogeneity and publication bias (‘file drawer bias’). In view of the high heterogeneity score in all variables (I^2^ > 75%, *p* < 0.001), we applied the random-effect model for the analysis. Publication bias was not detected in any of the time points (*p* > 0.05 by Egger’s test).

**Table 3:** Results of Meta-Analysis by Random Effect Model for Each Time Point.

Time Point	#Patients	n	Pooled Weighted Means		Heterogeneity		Publication Bias
			WM [95%CI][°C]	*p*-value	I^2^ (%)	*p*-value	Egger’s Test *p*
Pre-TKA	269	9	0.38 [0.17, 0.59]	0.0004	84	< 0.0001	0.088
Day 1	180	6	2.82 [1.74, 3.90]	< 0.0001	99	< 0.0001	0.965
Week 1	187	7	3.19 [2.52, 3.86]	< 0.0001	96	< 0.0001	0.54
Week 2	158	4	2.86 [1.92, 3.81]	< 0.0001	97	< 0.0001	0.118
Week 4–6	318	10	2.03 [1.92, 2.15]	< 0.0001	92	< 0.0001	0.339
Month 3	238	8	1.37 [0.70, 2.04]	< 0.0001	97	< 0.0001	0.557
Month 6	231	6	0.90 [0.38, 1.43]	0.0008	99	< 0.0001	0.375
Year 1	161	4	0.57 [0.10, 1.03]	0.016	94	< 0.0001	0.307

*n = number of studies; WM = weighted means of temperature difference between operated and non-operated knee in degrees Celsius (95% confidence intervals); Heterogeneity considered present if I^2^ > 50% and/or *p* < 0.1; Publication bias considered significant if Egger’s *p* < 0.05.

**Figure 5:**
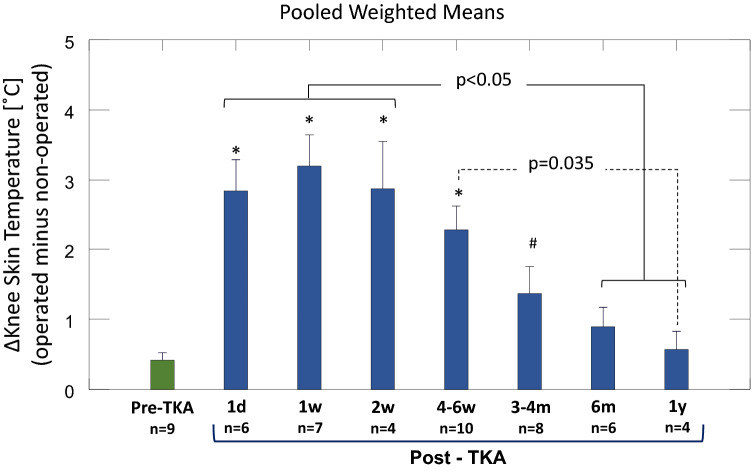
Knee Skin Temperature Before and After Total Knee Arthroplasty. Bar graph integrating meta-analysis results showing knee skin temperature differences in degrees Celsius before total knee arthroplasty (pre-TKA) and for each follow up time point (post-TKA) as well as the statistical results of the comparisons between these time-points. Bars = pooled weighted mean ± standard error of the mean by meta-analysis. Statistical results by 1-way ANOVA with Tukey as post-hoc test: *p < 0.005 vs Pre-TKA; #*p* = 0.015 vs 1 wk; TKA = total knee arthroplasty; n = number of studies included; d = day; w = week; m = months; y = year.

Figure 5 is an integrative bar graph of results from all time points with accompanying statistics. In uncomplicated knee recovery, ΔST was highest during the first 2-weeks post-surgery reaching a pooled mean of over 2.8 °C. The ΔST continued to be significantly higher than pre-TKA levels at 4–6 weeks (*p* < 0.0001). At 3-months post-TKA, ΔST was 1.4 °C and then gradually decreased to 0.9 °C and 0.6 °C at 6 and 12-months post-surgery respectively which were similar to pre-surgical levels (*p* > 0.95, pre-TKA vs. 6 and 12 months).

## Discussion

We reviewed and analyzed the quantitative profile of temporal changes in skin temperature after TKA in patients with uncomplicated recovery. There is a prominent increase in ΔST in the first 2 weeks post-TKA that gradually resolves by 6 months reaching near pre-surgical values at 1-year post-surgery when clinical recovery was considered satisfactory and to occur without complication.

The average ΔST prior to surgery (0.4 ± 0.1 °C) was very close to the threshold of minimal clinical important difference in symmetrical limbs (0.5 °C ^24^). This initial significant ΔST can be explained by the underlying knee osteoarthritis characterizing the majority of patients that arrive for TKA. Previous studies report that osteoarthritis causes elevated ST compared to the unaffected knee (0.1–0.6 °C)^8,25,26^. However, patients with bilateral osteoarthritis, or other bilateral pathologies such as rheumatoid arthritis, may not show this difference^27^. The increased skin temperature in the early weeks following TKA was expected due to the acute inflammation that normally accompanies surgical procedures. However, at 6 months, clinically significant differences in skin temperature (≥ 0.5 °C^24^) were still present and lingered 1 year post surgery. This may reflect ongoing healing with changes in patellar blood flow and/or skin microvasculature^28^ in the area of the surgery.

Studies of patients with delayed PJI following TKA, including some after surgical revisions, showed elevated ST. Mumingjiang et al. reported that patients with PJI at 6 months post-TKA had differences in ST between the operated and non-operated knees of 4.2 ± 1.0 °C versus 1.0 ± 0.3 °C in patients without PJI^16^. Romano et al. reported that patients with PJI 8 months after TKA had differences in ST of 1.6 ± 0.6 °C versus 0 ± 0.4 °C in patients without PJI^21^. In a subsequent study, Romano et al. reported that at 1 year after TKA, patients with PJI had differences in ST of 2.2 ± 1.5 °C versus 0.6 ± 0.5 °C in those without PJI^22^. The patients from those 3 studies were diagnosed with PJI on the basis of elevated serum inflammatory markers as well as clinical signs of infection including local redness and swelling. However, cases of chronic low-grade infection after TKA are not commonly associated with these local characteristics of inflammation, and are therefore sometimes misdiagnosed as aseptic failures^3,4^. Further studies are warranted to determine if skin temperature measurements are useful for this particularly challenging condition.

According to this meta-analysis, the average normal ΔST at 6 months after TKA is 0.9 °C, and, at 1 year, it is 0.6 °C. Neither is significantly different from the pre-surgical levels, and both are clearly lower than that found in the studies of patients with PJI. As determined by this meta-analysis, it appears that, for each time point, the acceptable differentiation between conditions should be based on the 95%CI upper limit of the pooled weighted means of uncomplicated patients with an addition of 0.5 °C^24^. For example, at 12 months (95%CI upper limit = 1.0 °C), patients with a difference in ST of > 1.5 °C may be at risk for PJI. This hypothesis should be confirmed in further studies.

Skin temperature can be measured in sufficient resolution with infrared thermometers or thermal imaging cameras^29^. In 2 of the reviewed studies, thermal imaging was used to determine the average temperature over the entire region surrounding the surgical area^15,16^. Others used infrared thermometers to measure skin temperature at specific points, some of which did not include the patella. Since the contralateral knee was evaluated as part of the outcome, the difference in instrumentation should not influence the overall results^30^. Likewise, although the position of the patient is expected to influence the absolute measurement because of differences in blood flow between postures, here too these differences are expected to be of limited influence since both knees were measured under similar conditions^31^. The factor that is considered to have the greatest influence is the ambient temperature. This has 2 components—the room temperature and the exposure time for acclimatization. It is reasonable to assume that orthopedic clinics are climate controlled to 25 °C or less, and that patients do not experience extreme cold or heat during visits^29^. The optimal skin acclimatization time for whole body thermograms was determined to be 10 min, but it is shorter for the lower limbs^32^. Moreover, in the latter study, the changes in ST during the acclimatization time were found to be parallel in symmetrical limbs. Thus, it can be assumed that 5 min will be sufficient to determine a difference of 1 °C or more between knees, although the exact time threshold should be determined in subsequent clinical studies.

Applying a tourniquet during the TKA procedure modifies the blood flow and therefore may affect knee ST. Alisi et al.^33^ reported that ST of the ankle region decreased on application of a tourniquet during a TKA procedure. However, ST levels returned to pre-procedural levels 20 min after tourniquet release. We are not aware of studies that reported long-term effects of temporary tourniquet application on knee ST.

Monitoring clinical status by ST is not a new concept. For example, monitoring foot ST has been included in the evidenced-based guidelines for prevention of diabetic foot ulcers by the International Working Group on the Diabetic Foot^34^. More recently, Ghosh et al. reported that monthly monitoring of foot ST may predict recurrence of diabetic foot ulcers^35^. Other medical applications of ST include monitoring skin flap viability and depth of burn wounds^36^.

Smartphone-based applications for monitoring patient condition post-arthroplasties are already available, and information from biosensors including infrared thermometers or thermal imaging cameras can be integrated easily. Measuring ST at home on a regular basis can detect possible deviations or irregularities that may provide important information of relevance for early diagnosis of evolving pathologies such as PJI.

Finally, the usefulness of skin temperature measurements for diagnosis of PJI relies on its ability to differentiate between normal and abnormal levels. After careful consideration, and as recommended by the GRADE workgroup criteria for certainty of evidence^37^, we estimate that the accumulated body of evidence presented in this meta-analysis is of high quality and is sufficient to represent the normal, temporal changes in ST following TKA in patients without complications.

### Limitations

In some studies, there were variations in patient posture, acclimatization, and region of interest (ROI). Nonetheless, they are of limited effect on outcomes since the operated and non-operated sides were subjected to similar conditions. Four studies included in this systematic-review did not report whether a tourniquet was used. Further studies are required to determine if peri-procedural tourniquet application has long-term effect on knee ST. Each study collected thermal information at different follow-up time-points which precluded a “paired” comparison. This required limiting the analysis to non-paired calculations which are more conservative.

## Conclusions

This systematic review and meta-analysis has permitted characterization of the temporal changes in ST in patients with uncomplicated recovery following TKA. Further studies of changes in skin temparature in patients with PJI at different time points post-TKA, with correlative clinical and microbiological data, will provide important additional information related to the diagnostic usefulness of this objective, non-invasive thermal imaging modality.

## Methods

### Guidelines and Registration

This systematic-review and meta-analysis was performed according to Preferred Reporting Items for Systematic Reviews and Meta-Analysis (PRISMA) guidelines^23^. The review question is stated according to PICO principles: Participants = patients with uncomplicated recovery following TKA; Intervention = thermography; Comparator = time points; Outcome = pooled weighted means of the difference between the operated and the non-operated knees (ΔST). The review protocol was registered prospectively in the international prospective register of systematic reviews, PROSPERO (CRD42021269864). The PRISMA-P checklist appears in the Supplementary file online.

### Search strategy

The searches were conducted using PubMed and EMBASE. The search targeted studies reporting anterior knee skin temperature of patients before and after unilateral TKA, without date or language restrictions, up to May 24, 2021. The search protocol was defined using the following keywords: ‘total knee arthroplasty’ or ‘total knee replacement’ and ‘thermography’ or ‘infra-red thermometry’ or ‘infrared imaging’ or ‘thermal imaging’ or ‘thermogram’ or ‘thermographic imaging.’ The specific search protocol for each database is found in the Supplementary Table S1, online. Additional references were identified through manual search in the included manuscripts as well as in relevant reviews and websites.

### Study eligibility

*Inclusion Criteria:* (1) adults before and after unilateral TKA; (2) uncomplicated recovery; (3) passive thermal measurements not following any physical activity or procedure other than TKA; (43) availability of data on the differences between operated and non-operated knee. *Exclusion Criteria*: (1) interventions other than TKA (i.e. injections, physical activity, other knee surgeries); (2) active thermal measurements (response to cryotherapy, exercise, heating) (3) patients not adults; (4) non-human studies; (5) language other than English; (6) groups treated with interventions that could affect inflammation; (7) studies with data of only the operated knee.

### Screening, study selection, and data extraction

The screening was performed by two reviewers (LG and OH) independently using the blind screening mode of RYYAN-QCRI software^38^. Studies were screened initially by title and abstract. The full text of studies was screened again according to eligibility criteria. Disagreements were settled by discussion or in consultation with a third author (SDG). The list of excluded studies appears in Supplementary Table S2, online.

Data extraction included: Study Descriptors—First author, year of publication, country of origin, data collection period; Study Population—sample size, male:female counts, age; TKA Procedure—surgical approach, use of tourniquet, type of prosthesis, whether the patella was replaced (a metal replacement may affect skin temperature), dates of follow up visits; Skin Temperature Measurement- details of measuring device, method, location (ROI), imaging setup, acclimatization period. Missing methodological information was completed by contacting corresponding authors. A difference in skin temperature between the operated and non-operated knees of 0.5 °C is considered the minimum clinically important difference in ST for the anterior knee^24^.

Study appraisal was conducted according to the quality of measurement methodology^26^ and included equipment resolution, environmental conditions during data collection, and ROI. Accordingly, the quality of each study was scored as good, fair, or poor considering availability of information on patient position, skin exposure time, or ROI (Table 2). None of the articles included in this meta-analysis was graded as ‘poor’.

### Data analysis and statistics

The primary study outcome for the quantitative analysis of ΔST was the pooled, weighted mean with 95% confidence intervals (CIs) as calculated by meta-analysis with a random-effect model. Meta-analyses were performed separately for each time point (i.e. pre-TKA and post-TKA at 1 day; 1,2, and 6 weeks; and at 3, 6, and 12 months). Heterogeneity between studies was assessed with I^2^ statistics (*p* < 0.1 and/or I^2^ > 50% indicated significant heterogeneity). Publication bias (‘file drawer bias’) was assessed by Begg’s Funnel-Plot and pseudo-95%-confidence limits. Egger’s test was used to determine bias. Comparisons between time points were performed using 1-way ANOVA with Tukey as the *post-hoc* test. Missing standard deviations were imputed by weighted average at each time point. When data were reported separately for the operated and non-operated knee, the average difference was calculated, and the SD was pooled. The Comprehensive Meta-Analysis (CMA) Version-3 software (Borenstein, M., Hedges, L., Higgins, J., & Rothstein, H. Biostat, Englewood, NJ 2013) and SYSTAT, version 13 (Systat Software, Chicago, USA) were used as appropriate for analyzing the data.

## Supplementary Information

Supplementary Information 1.

Supplementary Information 2.

## Data Availability

All data can be found in the tables supplied as part of the manuscript.
