# Supplementary material for: Natural history of changes in knee skin temperature following total knee arthroplasty: a systematic review and meta-analysis
Source: Sci Rep. 2023 Apr 26;13:6810. doi: 10.1038/s41598-023-33556-7 (PMC10133306; doi:10.1038/s41598-023-33556-7)
Supplement: Supplementary file 2 — Supplementary Information 2. [file 41598_2023_33556_MOESM2_ESM.docx]

**Natural History of Changes in Knee Skin Temperature Following Total Knee Arthroplasty - A Systematic Review and Meta-Analysis**

**SUPPLEMENTAL INFORMATION**

Table S1: Search Protocols

| **Database** | **Search Protocol** |
| --- | --- |
| **PubMed** | (("knee"[Title/Abstract] OR "arthroplasty, replacement, knee"[MeSH Terms]) AND ("temperature mapping"[Title/Abstract] OR "Thermography"[Title/Abstract] OR "infra red imaging"[Title/Abstract] OR "infra-red thermometry"[Title/Abstract] OR "infrared imaging"[Title/Abstract] OR "infrared scanning"[Title/Abstract] OR "ir imaging"[Title/Abstract] OR "IRT"[Title/Abstract] OR "thermal imaging"[Title/Abstract] OR "thermogram"[Title/Abstract] OR "thermographic imaging"[Title/Abstract] OR "thermology"[Title/Abstract] OR "Thermography"[MeSH Terms])) NOT ("Animals"[MeSH Terms] NOT ("Animals"[MeSH Terms] AND "Humans"[MeSH Terms])) |
| **EMBASE** | ((knee:ti,ab,kw OR 'knee surgery'/exp) AND (('temperature mapping':ti,ab,kw OR thermography:ti,ab,kw OR 'infra-red imaging':ti,ab,kw OR 'infra-red thermometry':ti,ab,kw OR 'infrared imaging':ti,ab,kw OR 'infrared scanning':ti,ab,kw OR 'ir imaging':ti,ab,kw OR irt:ti,ab,kw OR 'thermal imaging':ti,ab,kw OR thermogram:ti,ab,kw OR 'thermographic imaging':ti,ab,kw OR thermology:ti,ab,kw) OR 'thermography'/exp)) NOT ('animals'/exp NOT 'humans'/exp) |

Table S2: Excluded Studies with Reasons for Exclusion*

| **Author (Year)** | **Title** | **Reason for Exclusion** | **Detail** |
| --- | --- | --- | --- |
| Sadoghi (2009)[1] | Microwave Thermography as a Noninvasive Analysis of Anterior Knee Pain with Total Knee Prosthesis | Not retrieved |  |
| Glehr (2011)[2] | Thermal Imaging as a Noninvasive Diagnostic Tool for Anterior Knee Pain Following Implantation of Artificial Knee Joints | Wrong population | Grouped by pain and not by time point |
| Nica (2011)[3] | Pain Management as Part of the Complex Rehabilitation Treatment in Patients with Hip and Knee Arthroplasty | Additional intervention | 2 weeks of physiotherapy |
| Tanavalee (2011)[4] | Inflammation Related to Synovectomy During Total Knee Replacement in Patients with Primary Osteoarthritis | Wrong outcome | Not included in MA: Only skin temperature of operated knee |
| Ammer (2012)[5] | Temperature of the Human Knee - A Review | No new information | Systematic review |
| Romano (2012)[6] | Telethermographic Findings after Uncomplicated and Septic Total Knee Replacement | No new information | Results were presented in the 2011 manuscript and used here as control |
| Romano (2013)[7] | Value of Digital Telethermography for the Diagnosis of Septic Knee Prosthesis: A Prospective Cohort Study | Wrong population | Not uncomplicated patients |
| Calin (2015)[8] | A Review of the Effectiveness of Thermal Infrared Imaging in the Diagnosis and Monitoring of Knee Diseases | No new information | Review |
| Windisch (2016)[9] | Regional Differences in Temperature Course after Knee Arthroplasty | Wrong population | Measurement not over anterior aspect of knee |
| Miscuks (2017)[10] | Post-Operative Analgesia Level Assessment after Total Knee Replacement Evaluated by Infrared Thermography | Follow up < 1 day | Presented only in abstract form |
| Windisch (2017)[11] | Effects of Kinesio Taping Compared to Arterio-Venous Impulse Systemג„¢ on Limb Swelling and Skin Temperature after Total Knee Arthroplasty | Additional intervention | Kinesio-taping, arterio-venous impulse system |
| Lin (2018)[12] | Temporal Change of Interleukin-6, C-Reactive Protein, and Skin Temperature after Total Knee Arthroplasty Using Triclosan-Coated Sutures | Wrong outcome | Not included in MA: Only skin temperature of operated knee |
| Friedrich (2020)[13] | Thermography in Persistent Postoperative Pain after Knee Surgery | Wrong population | Patients grouped as hypothermic and hyperthermic |
| Scheidt (2020)[14] | A Systematic Review on the Value of Infrared Thermography in the Early Detection of Periprosthetic Joint Infections | No new information | Systematic review |
| Alisi (2021)[15] | Thermographic Assessment of Reperfusion Profile Following Using a Tourniquet in Total Knee Arthroplasty: A Prospective Observational Study | Follow up < 1 day |  |

*In order of publication year

**Excluded Studies**

1 Sadoghi, P. *et al.* Microwave thermography as a noninvasive analysis of anterior knee pain with total knee prosthesis. *J. Bone Joint Surg. Br.* **91 B**, 468 (2009).

2 Glehr, M. *et al.* Thermal Imaging as a Noninvasive Diagnostic Tool for Anterior Knee Pain Following Implantation of Artificial Knee Joints. *International Journal of Thermodynamics* **14**, doi:10.5541/ijot.334 (2011).

3 Nica, S. A., Mologhianu, G., Mitoiu, B. I. & Miron, L. S. F649 Pain management as part of the complex rehabilitation treatment in patients with hip and knee arthroplasty. *European Journal of Pain Supplements* **5**, 183-183, doi:<https://doi.org/10.1016/S1754-3207(11)70629-0> (2011).

4 Tanavalee, A., Honsawek, S., Rojpornpradit, T., Sakdinakiattikoon, M. & Ngarmukos, S. Inflammation related to synovectomy during total knee replacement in patients with primary osteoarthritis: a prospective, randomised study. *J Bone Joint Surg Br* **93**, 1065-1070, doi:10.1302/0301-620X.93B8.26719 (2011).

5 Ammer, K. Temperature of the human knee - A review. *Thermology International* **22**, 137-151 (2012).

6 Romanò, C. L., Logoluso, N., Dell'Oro, F., Elia, A. & Drago, L. Telethermographic findings after uncomplicated and septic total knee replacement. *Knee* **19**, 193-197, doi:10.1016/j.knee.2011.02.012 (2012).

7 Romanò, C. L. *et al.* Value of digital telethermography for the diagnosis of septic knee prosthesis: a prospective cohort study. *BMC Musculoskelet Disord* **14**, 7, doi:10.1186/1471-2474-14-7 (2013).

8 Calin, M. A., Mologhianu, G., Savastru, R., Calin, M. R. & Brailescu, C. M. A review of the effectiveness of thermal infrared imaging in the diagnosis and monitoring of knee diseases. *Infrared Physics & Technology* **69**, 19-25, doi:<https://doi.org/10.1016/j.infrared.2015.01.013> (2015).

9 Windisch, C., Brodt, S., Roehner, E. & Matziolis, G. Regional differences in temperature course after knee arthroplasty. *Knee Surg Sports Traumatol Arthrosc* **24**, 2686-2691, doi:10.1007/s00167-015-3809-z (2016).

10 Miscuks, A., Golubovska, I., Januskevica, V. & Zadoroznijs, S. in *36th Annual European Society of Regional Anaesthesia & Pain Therapy (ESRA) Congress* Vol. 42 e179 (Regional Anesthesia and Pain Medicine, Lugano, Switzerland, 13 – 16 September 2017, 2017).

11 Windisch, C., Brodt, S., Röhner, E. & Matziolis, G. Effects of Kinesio taping compared to arterio-venous Impulse System™ on limb swelling and skin temperature after total knee arthroplasty. *Int Orthop* **41**, 301-307, doi:10.1007/s00264-016-3295-z (2017).

12 Lin, S. J. *et al.* Temporal Change of Interleukin-6, C-Reactive Protein, and Skin Temperature after Total Knee Arthroplasty Using Triclosan-Coated Sutures. *Biomed Res Int* **2018**, 9136208, doi:10.1155/2018/9136208 (2018).

13 Friedrich, D. & Köhne, M. Thermography in persistent postoperative pain after knee surgery. *Orthopaedic Journal of Sports Medicine* **8**, doi:10.1177/2325967120S00294 (2020).

14 Scheidt, S. *et al.* A Systematic Review on the Value of Infrared Thermography in the Early Detection of Periprosthetic Joint Infections. *Z Orthop Unfall* **158**, 397-405, doi:10.1055/a-0969-8675 (2020).

15 Alisi, M. *et al.* Thermographic Assessment of Reperfusion Profile Following Using a Tourniquet in Total Knee Arthroplasty: A Prospective Observational Study. *Med Devices (Auckl)* **14**, 133-139, doi:10.2147/mder.S300726 (2021).
